# Weight Status, Psychosocial Factors, and Health-Related Quality of Life Among Jordanian Adolescents: A Cross-Sectional Study Using Structural Equation Modeling

**DOI:** 10.3390/children12091199

**Published:** 2025-09-08

**Authors:** Randa AlQaisi, Walid Al-Qerem, Belal Al-Zu’bi, Basil Al-Tah, Moath H. Daher, Mu’taz I. Alfreahat, Nasser A. Mousa, Seif D. Jankhout, Ansam O. Atrooz, Judith Eberhardt

**Affiliations:** 1 Department of Pediatrics, Faculty of Medicine, Mutah University, AlKarak 61710, Jordan; randasq@mutah.edu.jo; 2 Department of Pharmacy, Faculty of Pharmacy, Al-Zaytoonah University of Jordan, Amman 11733, Jordan; waleed.qirim@zuj.edu.jo; 3 Department of Special Surgery, Faculty of Medicine, Mutah University, AlKarak 61710, Jordan; belal88@mutah.edu.jo; 4 Jordan University Hospital, Amman 13046, Jordan; b.altah@ihh.com.jo (B.A.-T.); motazfrehat37@gmail.com (M.I.A.); 5 Ministry of Health, Amman 11118, Jordan; mhdaher25@med.just.edu.jo (M.H.D.); nasser1assem1mousa@gmail.com (N.A.M.); jankhotsaif7@gmail.com (S.D.J.); 6 Alkarak Governmental Hospital, AlKarak 61710, Jordan; ansamatrooz@gmail.com; 7 Department of Psychology, School of Social Sciences, Humanities and Law, Teesside University, Borough Road, Middlesbrough TS1 3BX, UK

**Keywords:** adolescents, obesity, quality of life, bullying, mental health, SEM, physical activity

## Abstract

**Background/Objectives:** Childhood and adolescent obesity is a growing public health concern in Jordan, with implications for both physical and psychological well-being. While obesity’s physical effects are well-documented, less is known about its broader association with adolescents’ quality of life (QoL), particularly in Middle Eastern contexts. This study aimed to investigate the associations between weight status, psychosocial factors, and health-related quality of life (HRQOL) among Jordanian adolescents. This study aimed to investigate the associations between weight status, psychosocial factors, and HRQoL among Jordanian adolescents. **Methods:** A cross-sectional study was conducted with 719 adolescents (63.3% female) aged 11–18, recruited from public schools and pediatric clinics in three regions of Jordan. Data were collected using validated questionnaires assessing demographics, health behaviors, mental health (PHQ-9, GAD-7, SMFQ), and HRQoL (PedsQL 4.0). Structural equation modeling (SEM) was used to examine direct and indirect pathways predicting physical and psychological QoL. **Results:** Bullying emerged as a key mediator between weight status and QoL, particularly for physical well-being. Depression and anxiety were significant predictors of poorer psychological QoL. Positive school experience, academic performance, physical activity, and paternal education were positively associated with QoL. Nutritional habits were inversely associated with anxiety levels. Model fit indices supported the adequacy of both the physical and psychological QoL models. **Conclusions:** The findings highlight the interconnected relationship of weight-related stigma, school environment, and lifestyle behaviors on adolescent well-being. Multi-component, school-based interventions targeting bullying, physical activity, and mental health literacy may be effective in improving QoL in this population.

## 1. Introduction

Childhood and adolescent obesity are increasingly recognized as a critical public health issue worldwide, with rates rising across both high- and low-income countries. According to the World Health Organization (WHO), more than 390 million children and adolescents aged 5–19 were classified as overweight or obese in 2022, highlighting the need for effective intervention strategies [1]. For children and adolescents aged 5–19 years, the WHO defines overweight as a BMI-for-age greater than one standard deviation above the WHO growth reference median, and obesity as a BMI-for-age higher than two standard deviations above the WHO growth reference median [2,3].

In Jordan, as in many Middle Eastern countries, the prevalence of obesity among youth has risen significantly over the past decade, driven by shifting dietary patterns, decreased physical activity, and increased screen time [4].

Childhood and adolescent obesity are linked to a range of short- and long-term health complications. It increases the risk of respiratory conditions, orthopedic issues, cardiovascular disease, and metabolic disorders [5]. Beyond its physical consequences, obesity is also profoundly correlated with psychological well-being and quality of life [6]. Adolescents with obesity frequently experience stigmatization, bullying, social isolation, and low self-esteem—factors that can negatively affect academic performance, relationships, and overall mental health.

In addition to physical health risks, adolescent obesity has been strongly linked to social and emotional challenges. Peer victimization, particularly bullying related to body weight, is a well-documented consequence of obesity and contributes to increased psychological distress in affected youth [7,8]. Bullying can result in lower self-esteem, school disengagement, and reduced participation in physical activity, which may further compromise both physical and mental health outcomes [9,10].

Obesity in adolescence is also associated with elevated rates of depression and anxiety [6,11]. Emerging evidence from both Western and Middle Eastern settings suggests that these internalizing symptoms can mediate the relationship between obesity and reduced quality of life and may persist into adulthood if not addressed early [6,12]. Understanding the pathways through which psychosocial factors influence health-related quality of life in this population is therefore critical for the development of effective, context-specific interventions.

Health-Related Quality of Life (HRQOL) is a multidimensional concept that encompasses physical, social, emotional, and academic well-being [13]. Assessing HRQOL in pediatric populations provides a comprehensive view of how chronic conditions like obesity affect daily functioning. Previous research has consistently shown that childhood and adolescent obesity is associated with significant reductions in HRQOL [11,14]. Children with obesity tend to experience greater psychological distress and lower overall well-being compared to their peers of healthy weight [11].

Despite the rising prevalence of pediatric obesity in Jordan, limited research has investigated its broader association with young people’s daily lives, particularly in terms of psychological and social well-being. Most existing studies in the region focus primarily on prevalence and physical health outcomes, with little attention given to how obesity affects quality of life [15]. Understanding these effects is critical, as quality of life reflects the lived experiences of youth and informs more holistic intervention efforts. This study aimed to address this gap by quantifying the prevalence of obesity among Jordanian children and adolescents and evaluating its association with their quality of life. By integrating physical and psychosocial measures, the study will generate context-specific evidence to support the design of more effective, culturally relevant prevention strategies in Jordan.

To better understand the multifaceted influences on adolescent well-being, this study is grounded in two complementary frameworks: the social-ecological model and the biopsychosocial model. The social-ecological model highlights how health and behavior are shaped by interactions between individuals and their environments, including peers, family, and institutional settings [16,17]. In the context of this study, factors such as bullying, parental education, and school experience are viewed as relational and environmental influences that can either protect or undermine adolescent well-being. The biopsychosocial model [18] further emphasizes the interplay between biological (e.g., weight status), psychological (e.g., depression, anxiety), and social (e.g., peer interactions) domains in determining health-related quality of life. These frameworks jointly informed the study design and support our use of structural equation modeling to examine complex, multidimensional pathways to adolescent health outcomes.

This study aimed to examine the relationships among weight status, bullying, psychosocial functioning (depression and anxiety), and HRQoL among Jordanian adolescents. In particular, we sought to determine whether bullying mediates the association between weight status and HRQoL, and whether depression and anxiety are associated with lower psychological HRQoL. We also examined the role of school experience, physical activity, academic performance, and paternal education in influencing HRQoL. Structural equation modeling was used to test both direct and indirect associations among these variables.

Based on prior research and the theoretical models underpinning this study, we proposed the following hypotheses:

**H1:**  *Higher weight status will be indirectly associated with lower physical and psychological quality of life, primarily mediated by experiences of bullying*.

**H2:**  *Greater levels of depression and anxiety, as measured by the PHQ-9 and GAD-7, will be associated with lower psychological quality of life*.

**H3:**  *Positive school experiences and higher academic performance will be positively associated with psychological quality of life*.

**H4:**  *Higher levels of physical activity will be positively associated with both physical and psychological quality of life*.

**H5:**  *Higher paternal education will be positively associated with quality of life, either directly or through academic performance and physical activity*.

## 2. Materials and Methods

### 2.1. Study Design and Participants

This study used a cross-sectional design to examine the relationships among weight status, quality of life, mental health symptoms, and social experiences in a sample of children and adolescents in Jordan. Participants aged 11–18 years were recruited from both pediatric clinics and public schools, representing a mix of clinical and community-based youth populations. The primary aim was to assess how various health and environmental factors are correlated with adolescents’ quality of life.

Eligible participants were Jordanian nationals within the target age range who were able to provide assent. Informed written consent was obtained from parents or legal guardians, followed by assent from the adolescents themselves. Individuals with severe cognitive impairments or communication difficulties that could interfere with valid questionnaire completion were excluded from the study.

The sociodemographic characteristics of the participants are presented in Table 1. A total of 719 children and adolescents participated in the study, with females comprising the majority (63.3%). The median age was 16 years (interquartile range: 14–16). Most participants reported that both their fathers (44.9%) and mothers (48.5%) had completed college or university education. In terms of weight status, the majority fell within the normal range (62.6%). Only 10.3% reported having a chronic disease. Regarding academic achievement, 47% of participants reported scores between 90 and 100. Lastly, the majority (72.3%) indicated they had never experienced bullying at school. The GAD-7 classified 15.9% of the sample in the severe anxiety group, and the SMFQ categorized 8.5% as severe depression. Cronbach’s alpha for GAD-7 and SMFQ were 0.90 and 0.76, respectively, indicating acceptable internal consistency for both questionnaires.

### 2.2. Sampling and Sample Size Justification

To capture a diverse range of backgrounds and experiences, a multistage cluster sampling approach was used. Three governorates were selected to represent the central, southern, and eastern regions of Jordan: Amman, Al-Karak, and Al-Zarqa. Within these governorates, public secondary schools were randomly selected to ensure school-level representation. In addition, participants were recruited from pediatric outpatient clinics at Al-Basheer Hospital, Prince Hamza Hospital, and Al-Karak Governmental Hospital. Including both clinical and educational settings allowed for a more comprehensive understanding of how weight and mental health issues manifest across different environments.

The sample size was computed based on established guidelines for structural equation modeling, which recommend a minimum of 5–10 participants per parameter [19], as the models in the present study included 45 estimated parameters, a minimum sample size of 225–450 participants was deemed appropriate. The final sample of the present study (N = 719) exceeds these thresholds. This substantially larger sample will enhance the reliability of subgroup analyses and accommodate potential non-responses or incomplete.

### 2.3. Ethical Considerations and Consent

Ethical approval for the study was obtained from the Ministry of Education in Jordan (3/10/53195 dated on: 23 October 2024) and from Mutha University (Ref#1492024 dated on: 10 October 2024). Written informed consent was obtained from the parents or legal guardians of all participants, and verbal assent was provided by the children prior to data collection. Participation was entirely voluntary, and participants were informed of their right to withdraw at any time without consequence. To ensure confidentiality, no identifying personal information was collected or stored.

### 2.4. Data Collection and Instruments

Data were collected between April and December 2024 using a structured questionnaire available in both digital and printed formats. In schools, the online survey link was distributed through official communication platforms and educational social media groups. In hospital settings, participants could complete the questionnaire on their own devices or using tablets provided by the research team. For school-based participants, height and weight measurements were obtained from health records to ensure accurate calculation of body mass index (BMI).

The questionnaire consisted of four main sections. The first section gathered demographic and lifestyle information, including age, sex, school grade, family income, and parental education levels. It also covered daily routines and health behaviors such as sleep duration, screen time, physical activity, mode of transportation to school, and dietary habits. Additionally, this section asked about medical conditions, medication use, past weight-related surgeries, and family history of overweight or obesity.

The second section focused on the school environment. Participants reported their academic performance using percentage categories, rated their overall satisfaction with school life, and indicated whether they had experienced bullying. If bullying had occurred, they were asked whether it was related to their body weight.

Mental health was assessed in the third section using three standardized and widely validated tools. The Patient Health Questionnaire-9 (PHQ-9) [20], modified for adolescents, includes nine items that assess depressive symptoms over the past two weeks. Each item is rated on a scale from 0 to 3, yielding a total score ranging from 0 to 27. Scores are categorized as follows: 0–4 (minimal depression), 5–9 (mild), 10–14 (moderate), 15–19 (moderately severe), and 20–27 (severe depression). Anxiety was measured using the Generalized Anxiety Disorder-7 (GAD-7) scale [21], which consists of seven items also rated from 0 to 3, producing a total score between 0 and 21. Severity levels are interpreted as 0–4 (minimal anxiety), 5–9 (mild), 10–14 (moderate), and 15–21 (severe). The Short Mood and Feelings Questionnaire (SMFQ) [22] was used to screen for depressive mood. This 13-item scale uses a 3-point response format (0 = not true, 1 = sometimes true, 2 = true), generating a total score between 0 and 26. A score of 12 or higher suggests the presence of significant depressive symptoms. These instruments were selected for their brevity, clinical relevance, and strong validity and reliability in adolescent populations [23]. The PHQ-9 and GAD-7 scale have both been translated into Arabic and culturally adapted to the Jordanian population [23].

The fourth section of the questionnaire assessed health-related quality of life using the Pediatric Quality of Life Inventory (PedsQL 4.0) Generic Core Scales [24]. The PedsQL was selected for its reliability, brevity, and strong clinical relevance in pediatric populations. This widely used and validated tool measures quality of life in children and adolescents across 23 items, divided into four core domains: Physical Functioning (8 items), Emotional Functioning (5 items), Social Functioning (5 items), and School Functioning (5 items). It provides a comprehensive assessment of physical, emotional, social, and academic well-being. This tool was also translated into Arabic and validated among children with and without chronic diseases in Jordan [25].

Participants aged 11 to 18 years completed the self-report version. Each item was rated on a 5-point Likert scale, ranging from 0 (“Never”) to 4 (“Almost Always”). For scoring, items were reverse scored and linearly transformed to a 0–100 scale, with higher scores indicating better perceived quality of life. Domain scores were calculated by averaging the scores of completed items within each subscale. A Physical Health Summary Score (based on the Physical Functioning scale) and a Psychosocial Health Summary Score (the average of Emotional, Social, and School Functioning scores) were computed. A Total Scale Score was also calculated by averaging all items. In accordance with standard guidelines, scores were not computed if more than 50% of items in a given scale were missing.

### 2.5. Statistical Analysis

SEM was conducted using the lavaan package (Version: 0.6-19) in R to explore the direct and indirect relationships among weight status, bullying, psychological and physical QoL, and other psychosocial variables. Analyses were performed using the Weighted Least Squares Mean and Variance adjusted (WLSMV) estimator, which is suitable for categorical and non-normally distributed data. Model fit was evaluated using multiple indices: the Comparative Fit Index (CFI), Tucker–Lewis Index (TLI), Root Mean Square Error of Approximation (RMSEA), and Standardized Root Mean Square Residual (SRMR).

Model development followed a stepwise approach, beginning with the inclusion of theoretically grounded predictors. Final models were refined by removing non-significant paths to ensure a parsimonious and interpretable structure. Modification indices were examined to guide potential model improvements.

Two separate SEM models were constructed: one predicting Physical QoL and the other predicting Psychological QoL. Both models included weight status, bullying, school experience, anxiety, depression, physical activity, and academic performance as key predictors.

## 3. Results

Participants’ responses to the PedsQL items are presented in Table 2. For both the physical and psychological domains, the majority of participants selected “Never” for most items. In the physical domain, the item with the highest proportion of “Never” responses was “It is hard for me to take a bath or shower by myself” (87%). In the psychological domain, the most frequently endorsed “Never” item was “It is hard to keep up when I play with other kids” (66.9%).

The median score for the physical domain was 84.38 (interquartile range: 71.88–93.75) out of a maximum of 100, with a Cronbach’s alpha of 0.85, indicating good internal consistency. The psychological domain had a median score of 80 (66.67–90), with a Cronbach’s alpha of 0.90, reflecting excellent reliability.

The correlation matrix (Table 3) revealed several statistically significant associations among the study variables. Weight status was positively correlated with bullying (r = 0.27, *p* < 0.001). Bullying was positively correlated with anxiety (r = 0.31, *p* < 0.001) and depression (r = 0.42, *p* < 0.001). School experience showed negative correlations with anxiety (r = −0.30, *p* < 0.001) and depression (r = −0.35, *p* < 0.001). Physical activity was positively correlated with Physical QoL (r = 0.38, *p* < 0.001) and negatively correlated with depression (r = −0.28, *p* < 0.001). Parental education and household income were positively correlated with academic performance (r = 0.26, *p* < 0.001 and r = 0.22, *p* < 0.001, respectively). Depression showed a strong negative correlation with Psychological QoL (r = −0.51, *p* < 0.001).

Model fit indices indicated that both the Physical QoL Model and the Psychological QoL Model demonstrated acceptable to good fit. The Physical QoL Model yielded χ^2^ (50) = 182.493, *p* < 0.001, with a CFI of 0.954 and a TLI of 0.978. The RMSEA was 0.061 (90% CI: [0.052, 0.071]), and the SRMR was 0.081. The Psychological QoL Model showed χ^2^ (53) = 198.765, *p* < 0.001, with CFI = 0.946 and TLI = 0.972. The RMSEA was 0.065 (90% CI: [0.056, 0.074]), and the SRMR was 0.083. All indices fell within acceptable ranges.

SEM identified several statistically significant direct predictors of physical QoL (Table 4). In the Physical QoL model, bullying was a strong negative predictor (β = −0.596, *p* < 0.001). Sex was positively associated with Physical QoL (β = 0.179, *p* < 0.001), as was physical activity (β = 0.165, *p* < 0.001). Paternal education level also showed a positive association with Physical QoL (β = 0.136, *p* = 0.002).

In the Psychological QoL model, bullying was a significant negative predictor (β = −0.144, *p* < 0.001). Depression level (β = −0.287, *p* < 0.001) and anxiety level (β = −0.368, *p* < 0.001) were also significant negative predictors. School experience (β = 0.069, *p* = 0.005), academic performance (β = 0.087, *p* < 0.001), physical activity (β = 0.068, *p* = 0.013), and paternal education level (β = 0.096, *p* = 0.031) were all positively associated with Psychological QoL.

Indirect predictors of physical and psychological QoL were also identified. For instance, weight status, which was significantly predicted by chronic disease (β = 0.164, *p* < 0.001), sex (β = 0.202, *p* < 0.001), age (β = −0.280, *p* < 0.001), and physical activity (β = −0.177, *p* < 0.001), was not found to directly predict QoL. However, it was a significant predictor of bullying (β = 0.323, *p* < 0.001), which in turn was associated with both physical and psychological QoL. Additionally, bullying (β = 0.683, *p* < 0.001) and chronic disease (β = 0.163, *p* = 0.001) significantly predicted both anxiety and depression levels.

## 4. Discussion

This study aimed to examine the relationships among weight status, mental health, school experience, and health-related quality of life (HRQoL) in a large sample of Jordanian adolescents, using both physical and psychological dimensions of well-being. Using structural equation modeling, the study identified key direct and indirect predictors of QoL, providing a detailed understanding of how psychosocial and behavioral factors interact in this population.

Findings from both models highlight the complex and interconnected nature of physical and psychological well-being among adolescents. Weight status did not directly predict physical QoL; rather, their association was mediated through bullying. The observed pathway, weight status → bullying → Physical QoL, suggests that the adverse physical health outcomes associated with obesity in adolescence may be partly socially driven. Adolescents with higher weight status were more likely to report experiences of bullying, which in turn was strongly associated with lower physical QoL. These findings reflect the broader psychosocial burden of obesity and align with a growing body of literature identifying peer victimization as a key mechanism through which weight-related stigma affects health [7,9]. Such results extend earlier work (e.g., Puhl & Latner, 2007; Griffiths et al., 2006) by confirming that these patterns remain highly relevant in contemporary adolescent populations [8,26].

These findings are consistent with the social-ecological model of health, which emphasizes the association between interpersonal and societal factors, including norms around body size, and individual outcomes [27]. In this context, bullying is not only a reflection of peer dynamics but also an expression of broader structural weight bias, which adolescents may internalize and which may manifest in reduced physical functioning or avoidance of physical activity. Recent studies suggest that such experiences can lead to somatic symptoms, diminished motivation to engage in physical exercise, and greater avoidance of public or peer-involved [10,28]. These findings point to the need for weight-inclusive school policies and anti-bullying initiatives as part of comprehensive strategies to support the physical well-being of adolescents with overweight or obesity.

Physical activity was positively associated with Physical QoL, echoing earlier findings that regular movement supports better physical functioning, cardiovascular health, and reduced physiological stress in adolescents [29,30]. Beyond physiological benefits, participation in physical activity may also enhance adolescents’ sense of competence, social engagement, and body satisfaction, all of which can contribute to improved perceptions of physical well-being. Recent research indicates that adolescents who perceive themselves as more physically active tend to report fewer psychosomatic symptoms, including fatigue and somatic complaints, even when overall lifestyle patterns are suboptimal [31]. These results point to the value of accessible, inclusive physical activity programs, especially for youth who may be at risk of physical health challenges due to social or environmental barriers.

Paternal education also showed a positive association with Physical QoL, aligning with the Family Investment Model, which proposes that parents with higher education are better positioned to provide health-promoting resources and environments [32]. In the Jordanian context, where social and economic roles within families are often shaped by traditional gender norms, paternal education may be correlated with adolescents’ engagement in health-promoting behaviors by affecting household priorities, access to resources, and parental modeling of lifestyle habits [33].

In addition, male participants reported higher Physical QoL than females. This finding is consistent with previous studies showing that adolescent girls are more likely to face body image concerns, internalized weight stigma, and social pressures related to appearance, all of which may affect their perceptions of physical health [34]. Girls may also encounter greater barriers to physical activity, including cultural expectations and reduced access to safe or socially acceptable exercise spaces. These gendered patterns suggest a need for tailored health promotion strategies that address the specific challenges girls face in maintaining physical well-being.

In the Psychological QoL model, both bullying and negative school experiences were associated with elevated levels of anxiety and depression, reinforcing a substantial body of research identifying school-based stressors as key contributors to adolescent mental health problems [35]. Peer victimization, in particular, was strongly linked to internalizing symptoms, supporting findings from longitudinal studies that show sustained exposure to bullying increases the risk for persistent psychological distress, including clinical depression and generalized anxiety [12,36]. These outcomes may be driven by disruptions in self-esteem, increased feelings of loneliness and social disconnection, and stress responses associated with repeated peer victimization—all of which have been linked to elevated risk for internalizing symptoms in adolescence [37,38,39].

Conversely, a positive school experience—including supportive teacher-student relationships, feelings of safety, and academic engagement—was associated with lower levels of anxiety and depression and directly predicted higher Psychological QoL. This protective effect aligns with Ecological Systems Theory, which positions schools as a critical part of the adolescent microsystem that can either buffer or intensify psychosocial risk depending on the quality of interactions and institutional culture [40]. A positive school climate can enhance psychological resilience by fostering a sense of belonging, stability, and competence—factors essential to emotional well-being during adolescence [41,42,43].

These findings suggest that mental health promotion in schools should go beyond anti-bullying campaigns to include broader efforts to cultivate inclusive, supportive, and engaging learning environments. Such interventions may have wide-reaching benefits for adolescent psychological health, particularly when tailored to address the diverse social challenges students face.

Nutritional habits were inversely related to anxiety, supporting evidence from the field of nutritional psychiatry, which explores how diet quality may be connected to mental health through mechanisms such as inflammation, oxidative stress, and gut–brain communication [44,45]. These findings suggest that nutrition should be considered a relevant, yet often overlooked, component in adolescent mental health interventions.

Depression was negatively associated with academic performance, consistent with the biopsychosocial model, which posits that emotional and cognitive symptoms, such as reduced concentration, low motivation, and fatigue, can interfere with learning and school engagement [46]. In contrast, academic performance showed positive associations with parental education and household income, aligning with established socioeconomic theories of educational attainment. These theories suggest that families with higher socioeconomic status (SES) are more likely to provide cognitively stimulating environments, academic resources, and stability that support academic achievement [47].

The association between physical activity and reduced depressive symptoms is consistent with the Behavioral Activation Model, which holds that engaging in goal-directed, rewarding activities can improve mood and reduce withdrawal behaviors [48]. Beyond behavioral pathways, physical activity has been shown to promote mental health through neurobiological effects, including increased endorphin release and improved sleep regulation, both of which are protective against depression [49]. Evidence from a recent systematic review and meta-analysis further supports the positive association between mental school-based physical activity interventions and mental health outcomes, including resilience, well-being, and anxiety symptoms in young people [50].

Together, these findings highlight the importance of integrated, school-based approaches to adolescent health. Interventions that combine academic support, physical activity, mental health awareness, and dietary education within a supportive school climate may be especially effective in promoting both physical and psychological quality of life. Given that schools provide a structured and accessible setting for preventive efforts, such programs hold considerable promise for improving adolescent outcomes at scale [51].

### Strengths, Limitations, and Future Directions

This study has several strengths. It is one of the few to examine the associations between obesity and quality of life among Jordanian youth using a large, regionally diverse sample drawn from both clinical and community settings. The inclusion of validated, widely used instruments enhances the reliability and comparability of the findings. The use of structural equation modeling allowed for the examination of both direct and indirect relationships among psychosocial, behavioral, and demographic variables.

However, the study also has limitations. Its cross-sectional design limits the ability to draw causal inferences about the relationships observed. Self-report measures, while practical and efficient, may be subject to response bias or social desirability effects. Additionally, although efforts were made to ensure broad regional representation, the findings may not be generalizable to all regions of Jordan or to adolescents outside the school or healthcare system. The exclusion of children with severe cognitive impairments or communication difficulties may also limit the applicability of results to more vulnerable populations.

Future research should adopt longitudinal designs to assess the directionality and stability of these associations over time. Incorporating objective measures of physical activity, nutrition, and health outcomes could further strengthen the validity of future findings. It would also be valuable to explore the potential moderating effects of resilience, peer support, and family functioning. Finally, intervention studies are needed to evaluate the effectiveness of school- and community-based programs targeting the key psychosocial and behavioral factors identified in this study.

## 5. Conclusions

This study examined how weight status, mental health, and school-related factors interact in relation to the quality of life of Jordanian adolescents. The findings suggest that the effects of obesity are not limited to physical health but also shape emotional and social experiences—particularly through mechanisms such as bullying and internalizing symptoms. Depression and anxiety were closely linked to poorer psychological well-being, while positive school experiences, higher physical activity, and greater parental education were associated with better outcomes.

These results point to the importance of addressing the broader context in which adolescents live, learn, and interact. Interventions that aim to improve quality of life should not focus solely on individual behaviors but also consider the role of school climate, peer relationships, and structural inequalities such as access to resources and education. In Jordan and similar settings, school-based programs that combine physical activity with academic and emotional support could offer a practical way to reach large numbers of young people.

Further research is needed to explore how protective factors such as family support and resilience are associated with the experience and management of weight-related stigma. Longitudinal studies would help clarify how these relationships evolve over time and inform more targeted prevention and support efforts.

## Figures and Tables

**Table 1:** Participants’ sociodemographic characteristics.

		Count (%) or Median (25–75 Percentiles)
Age		16 (14–16)
Gender	Female	455 (63.3%)
	Male	264 (36.7%)
What is the highest level of education your father has completed?	Less than high school	158 (22%)
	High school	238 (33.1%)
	College/University	323 (44.9%)
What is the highest level of education your mother has completed?	Less than high school	120 (16.7%)
	High school	250 (34.8%)
	College/University	349 (48.5%)
Weight status	Underweight	26 (3.6%)
	Normal	450 (62.6%)
	Overweight	123 (17.1%)
	Obesity	120 (16.7%)
Chronic diseases	No	645 (89.7%)
	Yes	74 (10.3%)
Income status	<300	139 (19.3%)
	300–500	232 (32.3%)
	501–1000	239 (33.2%)
	>1000	109 (15.2%)
Academic performance	<70	121 (16.8%)
	70–79	218 (30.3%)
	80–89	42 (5.8%)
	90–100	338 (47%)
How do you feel about your school experience?	Very negative	40 (5.6%)
	Negative	39 (5.4%)
	Neutral	254 (35.3%)
	Positive	310 (43.1%)
	Very positive	76 (10.6%)
Depression level	No or Minimal depression	196 (27.3%)
	Mild depression	214 (29.8%)
	Moderate depression	152 (21.1%)
	Moderately severe depression	96 (13.4%)
	Severe depression	61 (8.5%)
Anxiety level	Minimal anxiety	238 (33.1%)
	Mild anxiety	222 (30.9%)
	Moderate anxiety	145 (20.2%)
	Severe anxiety	114 (15.9%)
Depression score		8 (4–14)
Anxiety score		7 (3–12)
Have you experienced any form of bullying at school?	No	520 (72.3%)
	Yes	199 (27.7%)

**Table 2:** Participants’ item-level responses to the Pediatric Quality of Life Inventory (PedsQL) for both physical and psychological domains.

Items	Almost Always	Often	Sometimes	Almost Never	Never
	Count (%)				
Physical factor					
It is hard for me to walk more than one block	12 (1.7%)	30 (4.3%)	75 (10.7%)	142 (20.2%)	445 (63.2%)
It is hard for me to run	18 (2.6%)	39 (5.6%)	94 (13.5%)	171 (24.6%)	374 (53.7%)
It is hard for me to do sports activities or exercise	14 (2%)	35 (5%)	77 (10.9%)	149 (21.2%)	429 (60.9%)
It is hard for me to lift something heavy	15 (2.1%)	31 (4.4%)	102 (14.6%)	201 (28.7%)	351 (50.1%)
It is hard for me to take a bath or shower by myself	8 (1.1%)	15 (2.1%)	19 (2.7%)	49 (7%)	610 (87%)
It is hard for me to do chores around the house	21 (3%)	30 (4.3%)	68 (9.8%)	149 (21.4%)	429 (61.5%)
I hurt or ache	27 (3.9%)	55 (7.9%)	109 (15.6%)	220 (31.5%)	287 (41.1%)
I have low energy	37 (5.3%)	83 (11.8%)	126 (17.9%)	245 (34.9%)	212 (30.2%)
Psychological factor					
I feel afraid or scared	19 (2.7%)	56 (8%)	124 (17.8%)	188 (26.9%)	311 (44.6%)
I feel sad or blue	43 (6.1%)	85 (12.1%)	147 (21%)	215 (30.7%)	211 (30.1%)
I feel angry	47 (6.7%)	127 (18.2%)	170 (24.4%)	210 (30.1%)	143 (20.5%)
I have trouble sleeping	33 (4.7%)	73 (10.4%)	106 (15.1%)	189 (27%)	299 (42.7%)
I worry about what will happen to me	57 (8.1%)	84 (12%)	127 (18.1%)	177 (25.2%)	256 (36.5%)
I have trouble getting along with other kids	23 (3.3%)	39 (5.6%)	92 (13.1%)	155 (22.1%)	392 (55.9%)
Other kids do not want to be my friend	19 (2.7%)	31 (4.4%)	68 (9.7%)	174 (24.8%)	410 (58.4%)
Other kids tease me	12 (1.7%)	35 (5%)	61 (8.7%)	141 (20.2%)	450 (64.4%)
I cannot do things that other kids my age can do	16 (2.3%)	42 (6%)	55 (7.9%)	141 (20.2%)	444 (63.6%)
It is hard to keep up when I play with other kids	12 (1.7%)	29 (4.1%)	48 (6.9%)	143 (20.4%)	468 (66.9%)
It is hard to pay attention in class	23 (3.3%)	51 (7.3%)	142 (20.3%)	169 (24.1%)	316 (45.1%)
I forget things	42 (6%)	88 (12.6%)	178 (25.4%)	226 (32.3%)	166 (23.7%)
I have trouble keeping up with my schoolwork	13 (1.9%)	38 (5.4%)	82 (11.7%)	151 (21.6%)	415 (59.4%)
I miss school because of not feeling well	20 (2.8%)	30 (4.3%)	78 (11.1%)	190 (27.1%)	384 (54.7%)
I miss school to go to the doctor or hospital	17 (2.4%)	24 (3.4%)	71 (10.2%)	184 (26.3%)	403 (57.7%)

**Table 3:** Correlation matrix of the variables included in the study models.

	Age	Weight Status	Sex	Paternal Education	Maternal Education	Chronic Disease	Family Income	Academic Performance	School Experience	Depression Level	Anxiety Level	Bullying	Nutritional Practice	Physical Activity	Physical Activity	Psychological QOL	Overall Quality of Life
Age	1	−0.272 **	−0.366 **	−0.208 **	−0.253 **	−0.006	−0.206 **	−0.196 **	−0.207 **	0.229 **	0.231 **	−0.049	−0.129 **	−0.206 **	−0.091 *	−0.142 **	−0.127 **
Weight status	−0.272 **	1	0.193 **	0.003	0.049	0.152 **	0.033	−0.058	−0.026	0.070	0.022	0.264 **	0.030	−0.060	−0.118 **	−0.099 **	−0.120 **
Sex	−0.366 **	0.193 **	1	0.380 **	0.403 **	−0.019	0.421 **	0.185 **	0.152 **	−0.271 **	−0.213 **	0.037	0.135 **	0.131 **	0.220 **	0.227 **	0.247 **
Paternal Education	−0.208 **	0.003	0.380 **	1	0.588 **	−0.060	0.531 **	0.229 **	0.087 *	−0.166 **	−0.156 **	0.030	0.106 **	−0.027	0.206 **	0.215 **	0.231 **
Maternal Education	−0.253 **	0.049	0.403 **	0.588 **	1	−0.029	0.495 **	0.230 **	0.052	−0.189 **	−0.169 **	0.025	0.059	−0.028	0.153 **	0.228 **	0.208 **
Chronic Disease	−0.006	0.152 **	−0.019	−0.060	−0.029	1	−0.021	−0.040	−0.027	0.130 **	0.117 **	0.160 **	−0.058	−0.003	−0.125 **	−0.093 **	−0.118 **
Family Income	−0.206 **	0.033	0.421**	0.531 **	0.495 **	−0.021	1	0.221 **	0.016	−0.152 **	−0.113 **	−0.011	0.082 *	−0.061	0.171 **	0.141 **	0.170 **
Academic Performance	−0.196 **	−0.058	0.185 **	0.229 **	0.230 **	−0.040	0.221 **	1	0.130 **	−0.168 **	−0.108 **	−0.037	0.067	0.076 *	0.170 **	0.221 **	0.210 **
School Experience	−0.207 **	−0.026	0.152 **	0.087*	0.052	−0.027	0.016	0.130 **	1	−0.433 **	−0.394 **	−0.207 **	0.109 **	0.277 **	0.302 **	0.424 **	0.398 **
Depression Level	0.229 **	0.070	−0.271 **	−0.166 **	−0.189 **	0.130 **	−0.152 **	−0.168 **	−0.433 **	1	0.747 **	0.308 **	−0.168 **	−0.255 **	−0.539 **	−0.717 **	−0.687 **
Anxiety Level	0.231 **	0.022	−0.213 **	−0.156 **	−0.169 **	0.117 **	−0.113 **	−0.108 **	−0.394 **	0.747 **	1	0.276 **	−0.177 **	−0.203 **	−0.482 **	−0.692 **	−0.641 **
Bullying	−0.049	0.264 **	0.037	0.030	0.025	0.160 **	−0.011	−0.037	−0.207 **	0.308 **	0.276 **	1	−0.060	−0.083 *	−0.223 **	−0.347 **	−0.313 **
Nutritional Practice	−0.129 **	0.030	0.135 **	0.106 **	0.059	−0.058	0.082 *	0.067	0.109 **	−0.168 **	−0.177 **	−0.060	1	0.153 **	0.086 *	0.143 **	0.124 **

* *p*-value < 0.05, ** *p-*value < 0.01.

**Table 4:** Standardized Path Coefficients and Significance Levels for Predictors of Psychological and Physical Quality of Life.

Predictor	Outcome	β	*p*-Value
Common pathway			
Chronic Disease	Weight Status	0.164	<0.001
Sex	Weight Status	0.206	<0.001
Age	Weight Status	−0.279	<0.001
Physical activity	Weight Status	−0.175	<0.001
Weight Status	Bullying	0.27	<0.001
Chronic Disease	Bullying	0.159	<0.001
Bullying	Anxiety Level	0.292	<0.001
Nutritional habits	Anxiety Level	−0.133	0.003
School Experience	Anxiety Level	−0.742	<0.001
Bullying	Depression Level	0.808	<0.001
Sex	Depression Level	−0.252	<0.001
Age	Depression Level	0.309	<0.001
Physical activity	Depression Level	−0.339	<0.001
School Experience	Depression Level	0.663	<0.001
Anxiety Level	School Feeling	2.237	<0.001
Depression Level	School Feeling	−2.719	<0.001
Paternal Education	Academic Performance	0.131	0.012
Maternal Education	Academic Performance	0.117	0.028
Income	Academic Performance	0.108	0.03
Depression	Academic Performance	−0.139	0.003
Physical model			
Bullying	Physical QoL	−0.596	<0.001
Sex	Physical QoL	0.179	<0.001
Physical activity	Physical QoL	0.165	<0.001
Paternal Education	Physical QoL	0.136	0.002
Psychological model			
Bulling	Psychological QoL	−0.144	<0.001
Sex	Psychological QoL	0.04	0.245
Physical activity	Psychological QoL	0.068	0.013
Paternal Education	Psychological QoL	0.096	0.031
Depression	Psychological QoL	−0.287	<0.001
Anxiety level	Psychological QoL	−0.368	<0.001
School Experience	Psychological QoL	0.069	0.005
Academic Performance	Psychological QoL	0.087	<0.001

## Data Availability

The dataset supporting the conclusions of this article is available in the Zenodo repository: https://doi.org/10.5281/zenodo.15349165 (accessed on 6 May 2025).

## Abbreviations

The following abbreviations are used in this manuscript:

QOL	Quality of life
HRQOL	Health-related quality of life
SEM	Structural equation modeling
WHO	World Health Organization
BMI	Body mass index
PHQ-9	Patient Health Questionnaire-9
GAD-7	Generalized Anxiety Disorder-7
SMFQ	Short Mood and Feelings Questionnaire
PedsQL	Pediatric Quality of Life Inventory
WLSMV	Weighted Least Squares Mean and Variance adjusted
CFI	Comparative Fit Index
TLI	Tucker–Lewis Index
RMSEA	Root Mean Square Error of Approximation
SRMR	Standardized Root Mean Square Residual
SES	Socioeconomic status
